# Use of neural networks to predict vault values after implantable collamer lens surgery

**DOI:** 10.1007/s00417-021-05294-x

**Published:** 2021-07-27

**Authors:** Ke Xu, Xiaoxiao Liu, Yiming Lei, Hong Qi, Chun Zhang

**Affiliations:** 1 Department of Ophthalmology, Beijing Key Laboratory of Restoration of Damaged Ocular Nerve, Peking University Third Hospital, 49 North Garden Road Haidian District, Beijing, 100191 China; 2 Ministry of Education Engineering Research Center of Mobile Digital Hospital Systems, School of Electronics Engineering and Computer Science, Peking University, Beijing, 100871 China

**Keywords:** Vault, Implantable collamer lens, Neural network, Biometric

## Abstract

**Background:**

Appropriate sizing of the implantable collamer lens (ICL) and accurate prediction of the vault are crucial prior to surgery. However, sometimes, the vault value is higher or lower than predicted, necessitating reoperation. The present study aimed to develop neural networks for improving predictions of vault values following ICL implantation based on preoperative biometric data.

**Methods:**

This retrospective study included 137 eyes of 74 patients with ICLs. Linear regression and neural network analyses were used to examine the relationship between vault values at the 6-month follow-up and preoperative parameters (e.g., ICL characteristics and biometrics).

**Results:**

Linear regression analysis revealed that vault values were correlated with five variables: ICL size, anterior chamber depth (ACD), angle-to-angle (ATA), white-to-white (WTW), and lens thickness (LT) (adjusted R^2^ = 0.411). Inclusion of more input variables was associated with better performance in the neural network analysis. The degree of fit when all 11 variables were included in the neural network model was close to 1 (R^2^ = 0.98). R^2^ values for the quaternary neural network model enrolling four input variables (ICL size, ATA, ACD, and LT) reached 0.90.

**Conclusions:**

A neural network equation including the ICL size and biometric parameters of the anterior segment (ATA, ACD, and LT) can be used to predict the postoperative vault, aiding in the selection of an appropriate ICL size and reducing the need for reoperation after surgery.
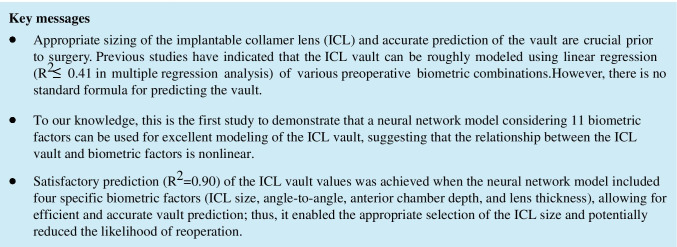

**Supplementary Information:**

The online version contains supplementary material available at 10.1007/s00417-021-05294-x.

## Background

The implantable collamer lens (ICL; STAAR Surgical, Nidau, Switzerland) is a type of phakic intraocular lens (IOL) used for the safe and effective correction of a wide range of refractive errors [1–4]. A safer ICL with a small central hole (V4c, KS-AquaPORT, STAAR Surgical AG) has recently been developed [5]. The distance between the center of the posterior artificial lens surface and the center of the anterior crystalline lens surface (hereafter referred to as the vault) plays an important role in the success of ICL surgery. Appropriate sizing of the ICL and accurate prediction of the vault are crucial prior to surgery. Lens sizing has traditionally been based on white-to white (WTW) and anterior chamber depth (ACD) measurements, as recommended by lens manufacturers. However, sometimes the vault value is higher or lower than predicted, necessitating reoperation.

Previous studies have indicated that the ICL vault can be roughly modeled using linear regression (R^2^ ≤ 0.41 in multiple regression analysis) of various combinations of preoperative biometrics [6–10]. However, to the best of our knowledge, there is no standard formula for predicting the vault, and no studies to date have thoroughly investigated the performance of all individual preoperative biometric factors and their combinations in predicting the ICL vault. Neural networks have enabled the mapping of complex nonlinear relationships in a variety of scientific domains. The present study aimed to utilize neural networks to comprehensively analyze the relationship between the ICL vault and preoperative biometric data.

## Methods

### Design

In this retrospective study, we reviewed the data of 137 eyes from 74 patients (sex, 16 men and 58 women) who underwent ICL implantation between May 2016 and May 2018 at the Department of Ophthalmology at Peking University Third Hospital (China); these patients were consecutively enrolled. The investigation was performed in accordance with the tenets outlined in the Declaration of Helsinki. The study protocol was approved by the Medical Science Research Ethics Committee of Peking University Third Hospital, which waived the requirement for informed consent because of the nature of the study (approval number: IRB00006761-M2020240).

Preoperative ophthalmologic examinations included tests of visual acuity and manifest refraction, slit-lamp examination, ophthalmoscopic examination, IOL Master examination (Carl Zeiss, Meditec AG, Jena, Germany), applanation A-scan ultrasound (OcuScan®, Alcon Inc., Fort Worth, TX), ultrasound biomicroscopy (UBM) (Paradigm Medical Industries, Salt Lake City, UT), and anterior segment optical coherence tomography (AS-OCT) (Visante Model 1000, Carl Zeiss Meditec).

### Patient data

The patients were included in the analysis if they met the following criteria: age, 21–45 years; ACD ≥ 2.80 mm; corneal endothelial cell count ≥ 2000 cells/mm^2^; stable refractive error ≥ 1 year; spectacle spherical power, –2.50 to –20.00 D; cylindrical power < 5.00 D; clear crystalline lens; no keratoconus findings on Belin–Ambrosio Enhanced Ectasia Display in Pentacam (Oculus, Wetzlar, Germany); no previous history of ocular pathology, ocular surgery, glaucoma, lens dislocation, or a diagnosed autoimmune or a connective tissue disease.

### Measurements

Preoperative angle-to-angle (ATA) distance, central corneal thickness, and ACD were determined via AS-OCT. The participants were seated with the mandible placed on the jaw bracket, forehead close to the frontal band, and eyes staring at the red light in front; the relevant parameters were measured under anterior chamber measurement mode with the QF value OK. Preoperative WTW distance was calculated as the average of the results obtained using calipers and IOL Master. The preoperative sulcus-to-sulcus (STS) distance was measured using UBM. UBM examinations were performed using an eyecup filled with methylcellulose solution after topical obucaine hydrochloride was instilled to anesthetize the cornea, while the participants were asked to fixate on a ceiling target and remain in the supine position. The examiner adjusted the probe perpendicular to the eyes, chose the UBM/STS pattern, and acquired the horizontal line images. All ATA, WTW, and STS measurements were horizontal. The ATA and WTW distances using calipers, WTW distance using IOL Master, and STS examinations were in a masked way without using cycloplegic drugs. Preoperative keratometry was performed using IOL Maser. The preoperative lens thickness (LT) was determined using A-scan ultrasonography. We performed A-scan examination after instilling topical obucaine hydrochloride to anesthetize the cornea, while the participants were seated and asked to fixate on a target in front. The examiner adjusted the probe perpendicular to the eyes, and all parameters were measured thrice. The average value of these measurements was calculated. The vault value was assessed 6 months postoperatively using AS-OCT.

### Surgical procedure

All surgeries were performed by one experienced surgeon (H.Q.). Following application of topical anesthesia, a 3-mm temporal clear corneal incision and a side hole were made, following which the anterior chamber was filled with a viscoelastic material (Hairont; Gallop Biological Products, Hangzhou, China). Then, V4c ICL was inserted through the corneal incision using an injector cartridge (STAAR Surgical AG). The ICL was placed in the posterior chamber, and the viscoelastic material was completely removed and replaced with a balanced salt solution, following which a miotic agent was instilled. All ICLs were placed in the horizontal posterior chamber.

### Calculation of lens power and diameter

Lens power and size were determined using the STAAR website (https://ocos.staarag.ch). The following preoperative parameters were entered: keratometry results, central corneal thickness, ACD, WTW, and refraction.

### Statistical and neural network analyses

Univariate linear regression and stepwise multiple regression analyses were performed to investigate the linear association between the vault value and preoperative variables (ICL size, ATA, ACD, LT, age, spherical equivalent [SE], WTW, STS, ICL optical power, and expected SE [ESE]). Statistical analysis was performed using SPSS for Windows, version 20.0 (IBM Corp., Armonk, NY, USA). A p value < 0.05 was considered statistically significant.

A neural network consists of an input layer connected to an input variable, one or more hidden layers, and an output layer that produces an output variable [11, 12]. The input layer of the neural network contained 11 independent variables: ICL size, ATA, ACD, LT, age, SE, WTW, STS, ICL optical power, ESE, and sex. The output layer of the neural network included the vault value.

The optimal settings for the hidden layer neurons were obtained via continuous iteration [13]. In general, the training process begins at a hidden layer, which is located between the input and output layers and performs nonlinear transformations of the inputs entered into the network, and the number of neurons increases until prediction error (PE) increases. Then, a second hidden layer is added to keep the number of parameters (weights plus deviations) approximately constant. The number of neurons per layer and the number of layers vary until good performance is achieved (i.e., p value < 0.05 and a minimum PE value). Given our small volume of data, we could perform multiple experiments to obtain different inputs that were suitable for different hidden layer network structures [14]. We selected two suitable hidden layer structures for each input. In the present study, our neural networks contained only one or two hidden layers.

Using this optimization process, the neural networks (NN) can be divided into the following five conditions:

- NN1: one input, one hidden layer with five nodes or two hidden layers with three nodes, one output;
- NN2: two inputs, one hidden layer with five nodes or two hidden layers with three nodes, one output;
- NN3: three inputs, one hidden layer with seven nodes or two hidden layers with five nodes, one output;
- NN4: four inputs, one hidden layer with seven nodes or two hidden layers with five nodes, one output;
- NN5: 11 inputs, one hidden layer with eight nodes or two hidden layers with six nodes, one output

The resulting index is the R^2^ (fitting degree) value, which reflects the fitting degree of the regression line to the observed value. R^2^ values are calculated using the following formula:

1 $${R}^2=\frac{\sum_{i=1}^n{\left({y}_i-\overline{y}\right)}^2\kern0.5em -{\sum}_{i=1}^n{\left({y}_i-\hat{y_i}\right)}^2}{\sum_{i=1}^n{\left({y}_i-\overline{y}\right)}^2}$$

In Eq. 1, $n,{y}_i,{\hat{y}}_i,\mathrm{and}\ \overline{y}$ refer to the number of regression points, measured value at point $i$, predicted value at point $i$, and mean value, respectively.

The range of R^2^ is [0,1]. Here, R^2^ is used to describe the degree of fit between the true and predicted values for the ICL vault. The fitted R^2^ value is obtained using Eq. 1. R^2^ values closer to 1 indicate a better degree of fit, indicating that the predicted ICL vault value is closer to the actual ICL vault value. In both the regression and neural network models, our goal was to obtain the predicted vault value. Moreover, we calculated the R^2^ values to examine how well the predictions approximated the real vault value. The optimal goodness of fit (R^2^) of this experiment was obtained by comparing the regression model with the neural network model results.

The learning rate controls the training progress of the model in the iterative process [15]. Following experimental verification, we observed that better results can be obtained by setting the learning rate to 0.1. The sigmoid function was used uniformly for activation functions in all models [16–18]. The optimization algorithm is a mathematical model that uses the idea of iteration to approximate the optimal solution to the problem. We chose the Adam optimization algorithm for the present study [19].

**Fig. 1:**
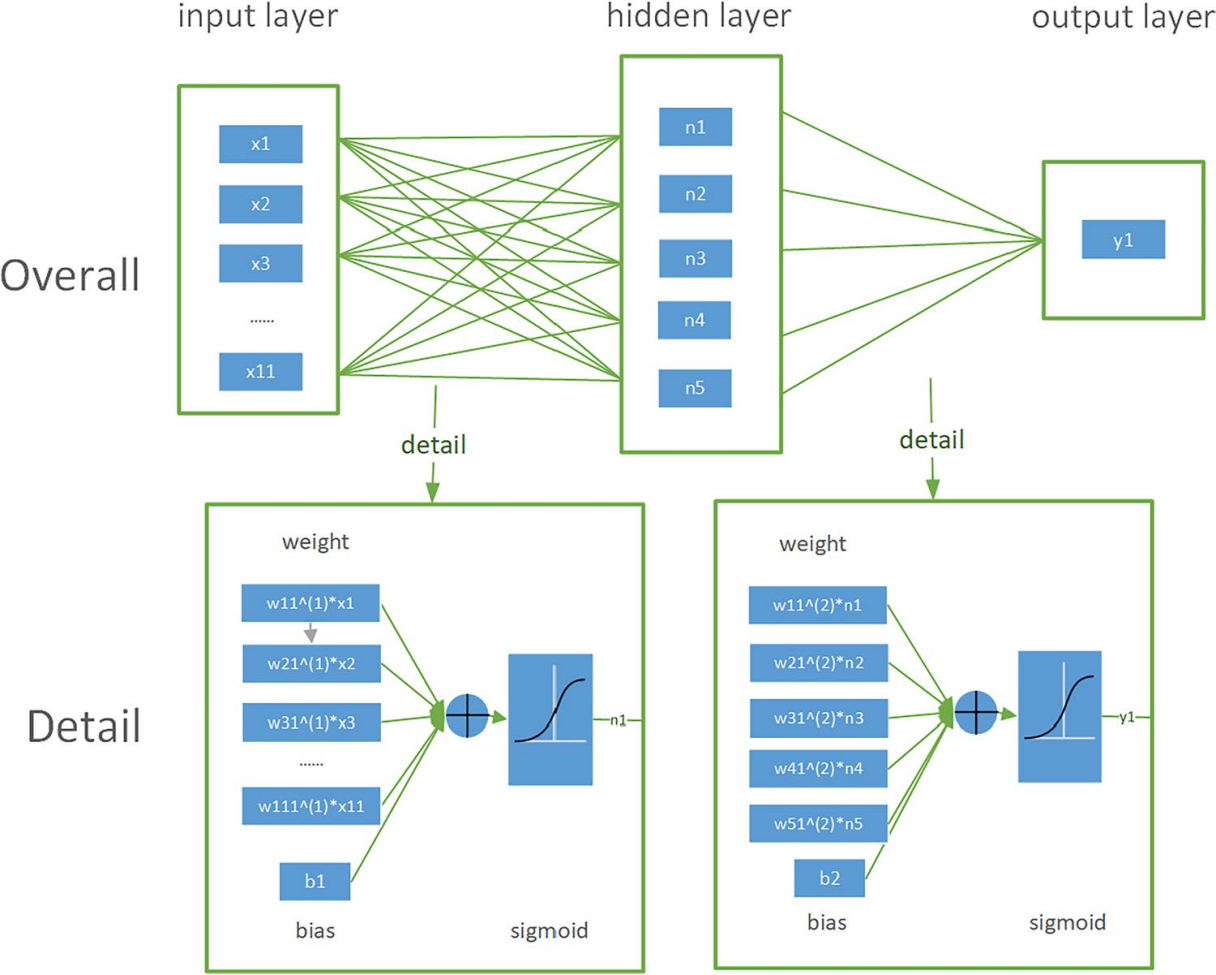
Detailed diagram of the neural network structure. The neural network consists of 11 nodes in one input layer, 5 nodes in one hidden layer, and 1 node in one output layer. The parameters × *1–* × *11*, *w*, *b*, *n*, and *y1* refer to the inputs, weight, deviation (i.e., a measure of the difference between the predictions of our model and the actual values), node in the hidden layer, and output, respectively. Full connection mode was adopted for the neural network. A sigmoid activation function was used for each layer

The structure of the neural network is presented in Fig. 1. The learning process can be summarized as follows: At the beginning of the learning process, the weights are set randomly, and a given set of inputs in the first layer is transmitted, following which the output data are generated. The neural network takes the loss function as the optimization objective to observe the optimal parameters. Each iteration of the neural network updates parameters based on loss function; thus, the faster the loss function changes, the larger the parameter update amplitude. The loss function reflects the difference between the actual and expected output data concerning the network error (mean square error).

When the neural network is modeled, the optimal model with the best result would be saved, along with all the parameters in the optimal model. Any surgeon can obtain the same results with the same data and can use these existing best models to test the new data to estimate the predicted vault.

The open-source Python library TensorFlow (Google Corp., Mountain View, CA) was used in PyCharm 2017.3.2 to design and train the neural network. TensorFlow is one of the most popularly used frameworks in deep learning [20]. Neural network structure, parameters, loss functions, and optimization algorithms can easily be defined using this library. All tests were run on laptops with the following specifications: Intel(R) Core (TM) i7-8550u CPU @1.80 GHz and 8 GB of RAM (Intel Corp., Santa Clara, CA).

## Results

### Descriptive statistics

In this study, we examined the data of 137 eyes from 74 patients (sex, 16 men and 58 women). Patient demographics, ICL characteristics, and biometric parameters of the anterior segment are presented in Table 1. The mean vault value 6 months after ICL implantation was 619.93 ± 245.66 μm (range, 190–1310 μm).

**Table 1:** Patient demographics, ICL characteristics, and biometric parameters of the anterior segment

Factors	Mean ± SD	Min	Max
Patients (eyes), n	74 (137)		
Sex (male/female), n	16/58		
Age (y)	32.61 ± 6.81	21	45
Spherical equivalent (D)	− 10.21 ± 4.34	− 25.00	− 2.25
Expected spherical equivalent (D)	− 0.53 ± 0.54	− 4.22	0.44
WTW (mm)	11.75 ± 0.37	11.00	12.50
ACD (mm)	3.23 ± 0.23	2.84	3.82
ATA (mm)	11.73 ± 0.42	10.54	12.67
STS (mm)	11.59 ± 0.51	10.46	12.80
LT (mm)	3.88 ± 0.29	3.28	4.76
ICL size (mm)	12.79 ± 0.40	12.10	13.70
ICL optical power (D)	− 10.41 ± 3.75	− 18.00	− 2.50
Vault (μm)	619.93 ± 245.66	190	1,310

*SD*, standard deviation; *WTW*, white-to-white; *ACD*, anterior chamber depth; *ATA*, angle-to-angle; *STS*, sulcus-to-sulcus; *LT*, lens thickness; *ICL*, implantable collamer lens

### Linear regression analysis

Univariate linear regression analysis was used to examine the linear relationship between each preoperative variable and the vault value. Our analysis identified seven of these factors as significant (ICL size, adjusted R^2^ = 0.243, *p* < 0.001; ACD, adjusted R^2^ = 0.146, *p* < 0.001; LT, adjusted R^2^ = 0.054, *p* = 0.004; ATA, adjusted R^2^ = 0.045, *p* = 0.007; WTW, adjusted R^2^ = 0.038, *p* = 0.013; STS, adjusted R^2^ = 0.036, *p* = 0.018; age, adjusted R^2^ = 0.033, *p* = 0.019).

Multiple stepwise regression analysis was performed using the postoperative vault as an explained variable and all preoperative parameters (age, sex, SE, ESE, WTW, ACD, ATA, STS, LT, ICL size, and ICL optical power) as explanatory variables. The best-fitting model included the following independent factors: ICL size, ACD, ATA, WTW, and LT. The model can be described as follows: postoperative vault (μm) = 57.5 × ICL size (mm) + 175.5 × ACD (mm) − 161.2 × ATA (mm) − 203.7 × WTW (mm) − 190.4 × LT (mm) − 2279.6 (R^2^ = 0.434, adjusted R^2^ = 0.411) (Supplementary Information 1).

### Neural network analysis

**Fig. 2:**
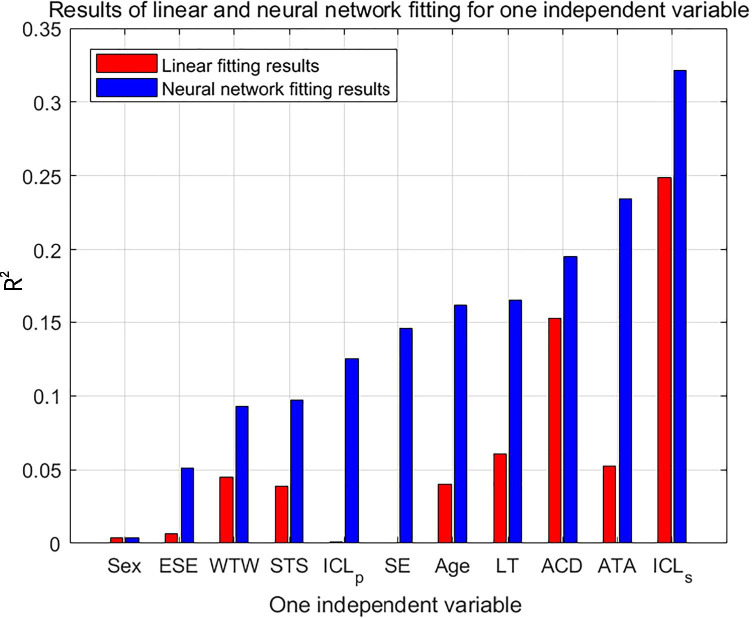
Results of linear and neural network fitting for one independent variable. The 11 preoperative variables are displayed on the x-axis, while R^2^ values are displayed on the y-axis. Red and blue bars represent linear and neural network fitting, respectively. ESE, expected spherical equivalent; WTW, white-to-white; ACD, anterior chamber depth; ATA, angle-to-angle; STS, sulcus-to-sulcus; LT, lens thickness; ICL_p_, optical power of the implantable collamer lens; SE, spherical equivalent; ICL_s_, size of the implantable collamer lens

Figure 2 depicts the results of 11 univariate fitting models for the linear and neural network analyses. As shown in the figure, the neural network results are better than the linear results. Analysis of R^2^ values revealed that ICL size was the most important parameter. Despite the differences in order, the first four optimal parameters (ICL size, ATA, ACD, and LT) were the same for the neural network and linear analyses.

**Fig. 3:**
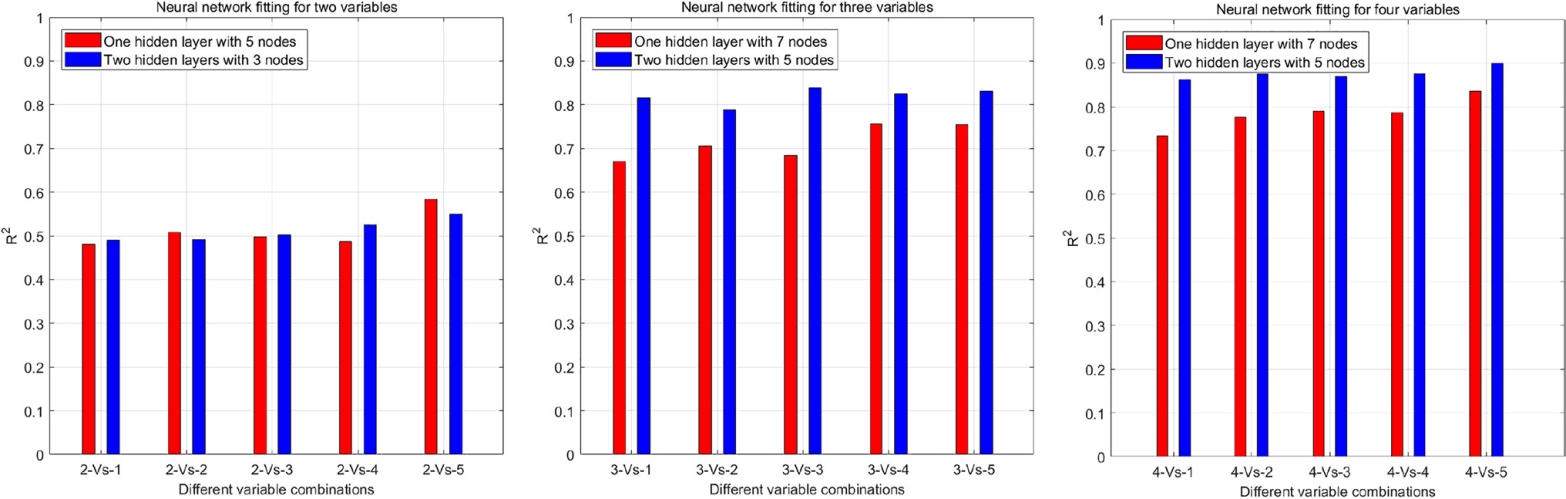
**a** Neural network fitting for two variables. X-axis: 2-vs-1: ICL size, STS; 2-vs-2: ICL size, WTW; 2-vs-3: ICL size, ACD; 2-vs-4: ICL size, LT; 2-vs-5: ICL size, ATA. R^2^ values are presented on the y-axis. Red and blue bars represent the model containing one hidden layer with five nodes and two hidden layers with three nodes in each layer, respectively. **b** Neural network fitting for three variables. X-axis: 3-vs-1: ICL size, ACD, WTW; 3-vs-2: ICL size, ATA, WTW; 3-vs-3: ICL size, ACD, LT; 3-vs-4: ICL size, ATA, LT; 3-vs-5: ICL size, ATA, ACD. R^2^ values are presented on the y-axis. Red and blue bars represent the model with one hidden layer and seven nodes and the model with two hidden layers and five nodes in each layer, respectively. **c** Neural network fitting for four variables. X-axis: 4-vs-1: ICL size, ATA, WTW, STS; 4-vs-2: ICL size, ACD, ATA, WTW; 4-vs-3: ICL size, ATA, LT, WTW; 4-vs-4: ICL size, ACD, LT, WTW; 4-vs-5: ICL size, ACD, ATA, LT. R^2^ values are presented on the y-axis. Red and blue bars represent the model with one hidden layer and seven nodes and the model with two hidden layers and five nodes in each layer, respectively. WTW, white-to-white; ACD, anterior chamber depth; ATA, angle-to-angle; STS, sulcus-to-sulcus; LT, lens thickness; ICL, implantable collamer lens

The results of the bivariate neural network analysis continued to improve for the first five combinations. ICL size was included in the five best combinations (Fig. 3a).

The results of the ternary neural network analysis continued to improve for the first five combinations (Fig. 3b). The best model (NN3, R^2^ = 0.84) contained three inputs (ICL size, ACD, ATA) and two hidden layers with five nodes, and had a sigmoid activation function.

Of the five outcomes for the quaternary neural network analysis, the second, third, and fourth results were similar (Fig. 3c). The parameters for the best combination were the same as those for the top four outcomes in the univariate model: ICL size, ACD, ATA, and LT. The worst model exhibited an R^2^ value > 0.73 for a small number of network layers (one hidden layer), while the best model exhibited an R^2^ value of 0.90. The best neural network model (NN4) exhibited the following characteristics: four inputs (ICL size, ACD, ATA, and LT), two hidden layers with five nodes, a sigmoid activation function, and an R^2^ value of 0.90. Part of the calculation for the optimal network structure is summarized in Supplementary Information 2.

**Fig. 4:**
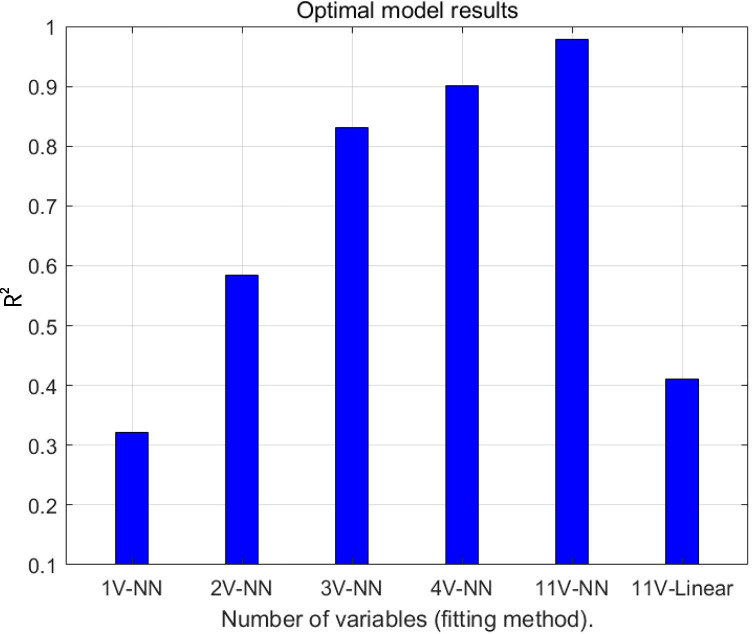
Optimal model results. The abscissa reflects the optimal combination of parameters for models with different numbers of variables under different fitting methods, where 1 V-NN represents the use of a single variable (ICL size). 2 V-NN: ICL size, ATA; 3 V-NN: ICL size, ACD, ATA; 4 V-NN: ICL size, ACD, ATA, and LT; 11 V-NN/11 V-Linear: age, sex, spherical equivalent, expected spherical equivalent, WTW, ACD, ATA, STS, LT, ICL size, ICL optical power. NN represents the neural network fitting model. R^2^ values are presented on the y-axis. WTW, white-to-white; ACD, anterior chamber depth; ATA, angle-to-angle; STS, sulcus-to-sulcus; LT, lens thickness; ICL, implantable collamer lens

As shown in Fig. 4, a greater number of input variables yielded a better result. When all 11 variables were included in the neural network model, R^2^ values were close to 1 (R^2^ = 0.98 in the model with two hidden layers and six nodes in each layer). In contrast, the ternary and quaternary neural network and linear regression models yielded R^2^ values of > 0.8, > 0.9, and < 0.5, respectively.

## Discussion

ICL implantation remains challenging because of difficulties in determining the appropriate lens size and predicting the vault value. Recent studies utilizing regression analyses have reported that the postoperative vault can only be explained in approximately 41.0% of cases [6–10]. In the present study, we performed linear regression and neural network analyses to examine the relationship between the preoperative biometric variables and the postoperative vault. To our knowledge, this is the first study to demonstrate that a neural network model considering 11 biometric factors can be used for excellent modeling of the ICL vault (R^2^ = 0.98 in the model), suggesting that the relationship between the ICL vault and biometric factors is nonlinear. Indeed, no previous studies have achieved such a strong fit. Satisfactory prediction of the ICL vault values was achieved when the neural network model included four specific biometric factors (ICL size, ATA, ACD, and LT; R^2^ = 0.90 in the model), allowing for efficient and accurate vault prediction.

Our multiple regression analysis yielded a result close to the highest linear fitting value reported in previous vault prediction studies (adjusted R^2^ = 0.411) [6–10]. However, linear regression analysis explained no more than half of the vault prediction. Confounding factors other than horizontal compression should be considered, including vertical compression by the iris, the dampening effect of ciliary sulcus structures, and the innate ICL lens vault [21, 22]. Indeed, clinical experience suggests that the ICL can be located at different positions in the posterior chamber, and the haptics may not be in the ciliary sulcus in most cases [23, 24]. These factors may not exhibit a simple linear correlation with the vault.

For our univariate network model, the top four R^2^ values were observed when ICL size, ACD, LT, and ATA were used. The same four variables yielded the best results in our linear regression analysis, and the main variable affecting the vault was the ICL size in both models (R^2^ = 0.24, *p* < 0.001 in linear regression analysis and R^2^ = 0.32 in univariate network analysis). The ICLs used in the present study (V4c ICL) are available in four sizes only: 12.1, 12.6, 13.2, and 13.7 mm. Our data indicated that the selected ICL size was the main factor influencing the postoperative vault.

For the ternary network model, the combination yielding the highest R^2^ value was that including the ICL size, ATA, and ACD. For the quaternary network model, the combination yielding the highest R^2^ value was that including the ICL size, ATA, ACD, and LT. R^2^ values for the next three ranked neural network models were almost identical (Fig. 3c). These three models included WTW rather than ATA, ACD, or LT. These findings indicated that the ICL size is the main factor affecting the postoperative vault, with strong contributions from ATA, ACD, and LT and more peripheral contributions from WTW.

Among the horizontal measurements obtained (ATA, WTW, STS), only ATA was included in the best quaternary neural network model (R^2^ values of approximately 0.90). Recent studies have highlighted the capability of AS-OCT for accurately calculating the ICL size based on ATA measurements [6, 25]. In one such study, multiple linear regression analysis determined that the ICL size and ATA values were the most important determinants of the postoperative vault (adjusted R^2^ = 0.41) [6]. In our bivariate network model, the combination yielding the highest R^2^ value also included the ICL size and ATA. Although ATA does not reflect the sulcus diameter, it may be more accurate and reliable than other biometric parameters measured in the horizontal dimension. This finding may be explained by the nature of AS-OCT, which enables automatic, non-contact measurement with high resolution and reproducibility.

In contrast to the traditional lens-sizing method, WTW was not included in the top quaternary neural network model. Given that WTW can be measured manually with calipers, there is a wide variation in the values obtained by different examiners [26]. WTW can also be acquired automatically using anterior segment imaging equipment. Nonetheless, the corneal limbus cannot be clearly visualized in a considerable number of cases, which may result in high variance and unsatisfactory reproducibility [26, 27].

In the present study, we observed no significant linear relationship between the horizontal STS distance and the vault value. Therefore, STS distance was removed from the list of explanatory variables in the multiple regression analysis. STS distance was also absent from the neural network models yielding the highest R^2^ values. Although various lens-sizing formulas have been developed based on UBM measurements of STS [28, 29], these UBM-based formulas have not been widely adopted by surgeons because of the invasiveness and low reproducibility of UBM [30]. A meta-analysis by Packer revealed that an STS-based formula was not superior to a WTW-based formula [31]. Additional factors may interfere with measurement, including the examiner error, ocular position, and anatomic variations in the ciliary sulcus.

Given that the current STAAR surgical calculator cannot predict the postoperative vault, the results of this study may aid in the prediction of vault values. Without the use of an additional software [9], the three values (ATA, ACD, and LT) and the ICL size, which were determined using the STAAR calculator, were used to predict the vault value. If the calculated vault value is not within the range of 250–1000 µm [10], the user can enter another relevant size into the model. If the calculated vault value is too large or too small, the user could choose a larger lens size and place it in the vertical posterior chamber, as STS increases closer to the vertical axis rather than the horizontal meridian [32].

The present study had some limitations, including its small sample size and retrospective design. Therefore, we could only train rather than test the neural network. Future prospective clinical studies involving multiple centers are required to optimize the model and help develop an ICL size calculator based on NN. Long-term changes in central vaulting because of accommodation or chronologic changes in crystalline LT must also be considered [4]. Further research is required to determine which vault range is safe and ideal in the long term.

In conclusion, the present study demonstrated that NN incorporating preoperative biometric data can be used to predict the postoperative vault. The equation generated using neural network analysis allows for precise vault prediction, thus enabling appropriate selection of the ICL size.

## Supplementary Information

Below is the link to the electronic supplementary material.

Supplementary file1 (DOCX 36 KB)

Supplementary file2 (DOCX 26 KB)

## Data Availability

The datasets used or analyzed during the current study are available from the corresponding author on reasonable request.

## Abbreviations

ACD: Anterior chamber depth
AS-OCT: Anterior segment optical coherence tomography
ATA: Angle-to-angle
ESE: Expected spherical equivalent
ICL: Implantable collamer lens
IOL: Intraocular lens
LT: Lens thickness
NN: Neural networks
SE: Spherical equivalent
STS: Sulcus-to-sulcus
UBM: Ultrasound biomicroscopy
WTW: White-to-white
