# Supplementary material for: Use of neural networks to predict vault values after implantable collamer lens surgery
Source: Graefes Arch Clin Exp Ophthalmol. 2021 Jul 27;259(12):3795–803. doi: 10.1007/s00417-021-05294-x (PMC8589809; doi:10.1007/s00417-021-05294-x)
Supplement: Supplementary file 1 — Supplementary file1 (DOCX 36 KB) [file 417_2021_5294_MOESM1_ESM.docx]

**Supplementary Information 1:**

**Scatterplot of postoperative vault and predicted vault values after performing multiple stepwise regression analysis**


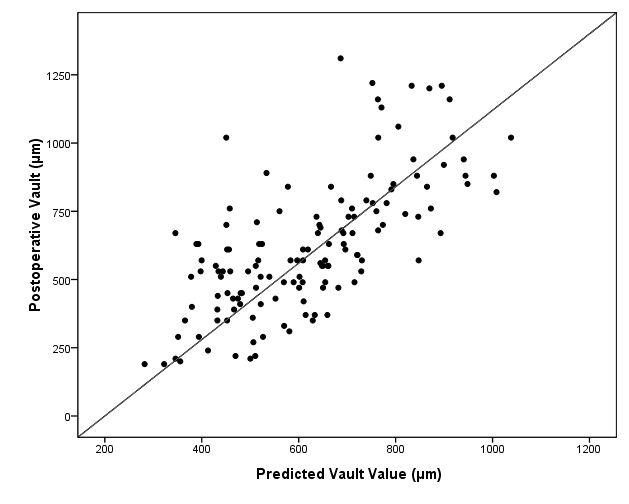


Correlation between the postoperative vault and predicted vault values determined using multiple stepwise regression analysis (postoperative vault [μm] =57.5 × ICL size [mm] + 175.5 × ACD [mm] – 161.2 × ATA [mm] – 203.7 × WTW [mm] – 190.4 × LT [mm] – 2279.6 [R^2^=0.434, adjusted R^2^=0.411]).

ICL, implantable collamer lens; ACD, anterior chamber depth; ATA, angle-to-angle; WTW, white-to-white; LT, lens thickness
