# Supplementary material for: Use of neural networks to predict vault values after implantable collamer lens surgery
Source: Graefes Arch Clin Exp Ophthalmol. 2021 Jul 27;259(12):3795–803. doi: 10.1007/s00417-021-05294-x (PMC8589809; doi:10.1007/s00417-021-05294-x)
Supplement: Supplementary file 2 — Supplementary file2 (DOCX 26 KB) [file 417_2021_5294_MOESM2_ESM.docx]

**Supplementary Information 2:**

Part of the calculation process for the optimal network structure can be summarized as follows:

$n11=g(w_{11}^{(1)}*x1+w_{21}^{(1)}*x2+w_{31}^{(1)}*x3+w_{41}^{(1)}*x4+b1)$ (2)

$n22=g(w_{12}^{\left( 2 \right)}*n11+w_{22}^{\left( 2 \right)}*n12+w_{32}^{\left( 2 \right)}*n13+w_{42}^{\left( 2 \right)}*n14+w_{52}^{\left( 2 \right)}*n15+b2)$ (3)

$y1=g(w_{11}^{\left( 3 \right)}*n21+w_{21}^{\left( 3 \right)}*n22+w_{31}^{\left( 3 \right)}*n23+w_{41}^{\left( 3 \right)}*n24+w_{51}^{\left( 3 \right)}*n25+b3)$ (4)

$g(z)=\frac{1}{1+e^{-z}}$ (5)

In these equations, *x1–x4* refers to the four input variables, *y1* refers to the output variable, *n11–n15* refers to the first hidden layer with five nodes, and *n21–n25* refer to the second hidden layer with five nodes. In $w_{ij}^{\left( n \right)}$, *i* represents the *i*^th^ input, *j* represents the *i*^th^ input to the *j*^th^ output, and *n* carries the same meaning as the number in the weight matrix, where$W^{(1)}$ represents the input to the first hidden layer’s weight matrix, $W^{(2)}$ represents the weight matrix between the two hidden layers, and $W^{(3)}$ represents the weight matrix for the second hidden layer to the output layer. In addition, *b1–b3* represent the deviation of each layer, while *g(z)* represents the sigmoid activation function.

Backpropagation was then performed while controlling for loss using the loss function, and the weight and deviation parameters were continuously optimized through multiple iterations of gradient descent. For the final, optimal model, the R^2^ between the predicted value of the ICL vault and the real value was 0.90.
